# Thermally induced gas flows in ratchet channels with diffuse and specular boundaries

**DOI:** 10.1038/srep41412

**Published:** 2017-01-27

**Authors:** Vahid Shahabi, Tobias Baier, Ehsan Roohi, Steffen Hardt

**Affiliations:** 1 High Performance Computing (HPC) Laboratory, Department of Mechanical Engineering, Ferdowsi University of Mashhad, 91775-1111, Mashhad, Iran; 2 Institute for Nano- and Microfluidics, Technische Universität Darmstadt, Alarich-Weiss-Straße 10, 64287 Darmstadt, Germany

## Abstract

A net gas flow can be induced in the gap between periodically structured surfaces held at fixed but different temperatures when the reflection symmetry along the channel axis is broken. Such a situation arises when one surface features a ratchet structure and can be augmented by altering the boundary conditions on different parts of this surface, with some regions reflecting specularly and others diffusely. In order to investigate the physical mechanisms inducing the flow in this configuration at various Knudsen numbers and geometric configurations, direct simulation Monte Carlo (DSMC) simulations are employed using transient adaptive subcells for collision partner selection. At large Knudsen numbers the results compare favorably with analytical expressions, while for small Knudsen numbers a qualitative explanation for the flow in the strong temperature inhomogeneity at the tips of the ratchet is provided. A detailed investigation of the performance for various ratchet geometries suggests optimum working conditions for a Knudsen pump based on this mechanism.

In a rarefied gas, the temperature field can play a significant role in inducing flow. According to the asymptotic theory for small Knudsen numbers and numerical observations based on kinetic theory, various types of flow can be induced. The portfolio includes thermal creep flow^1^,^2^,^3^,^4^,^5^, thermal stress slip flow^6^,^7^ and inverted thermal creep flow^8^,^9^ as well as (nonlinear) thermal stress flow^7^,^10^,^11^. The first three on the list are boundary effects, connecting the velocity distribution function of gas molecules in the bulk to their distribution at the wall within a thin Knudsen layer of thickness of the mean free path of gas molecules, while thermal stress flow occurs due to temperature induced stresses in the bulk, such that boundaries play a secondary role of prescribing the temperatures within the domain for this kind of flow.

In a simplified picture, thermal creep flow along a temperature gradient on a diffusely reflecting wall can be understood from the momentum in wall direction carried towards a patch of wall by gas molecules originating from regions of different temperature in the gas. Since molecules arriving from the warmer region carry a larger momentum than molecules originating from the colder side, a net momentum towards the colder side is imparted onto the wall from the gas. By momentum conservation, a corresponding force in opposite direction acts on the gas phase by the stationary wall and as a result, gas moves from the cold side to the hot side, with the corresponding flow being termed thermal creep or thermal transpiration.

Similarly, inverted transpiration is a boundary effect due to the different reflection properties of fast and slow particles at the wall. Here, fast molecules coming from the hot regions are reflected predominantly specularly, while slow molecules coming from the cold regions predominantly thermalize, reflect diffusively and thus impart a net tangential momentum to the wall^8^,^9^. As a result, a net force towards the hot region is imparted on the wall. In turn, the gas adjacent to the wall is driven toward the colder region.

Unlike thermal creep flow, thermal stress slip flow along a wall is induced in regions where temperature isolines are not parallel to each other and can occur even at walls with uniform temperature. The flow is in the direction opposite to the thermal creep, i.e. towards the colder region. However, its magnitude is of second order in temperature derivatives and when the wall is not isothermal it is thus usually overshadowed by thermal creep, which is proportional to the thermal gradient.

The lowest order contributions to thermal stresses in the bulk of the gas are a linear part proportional to the second derivatives of the temperature field and a nonlinear part of second order in the temperature gradients. As Epstein pointed out^12^, when the temperature field is governed by the Laplace equation the linear part will not constitute a net force on the gas and an induced flow will thus be nonlinear in the temperature. Thus again, when present, thermal creep flow will usually dominate in situations of small temperature variation.

Two other kinds of thermal flows which are induced around sharp corners are thermal edge flow and radiometric flow. If the boundary of a uniformly heated plate has a sharp edge, a flow called thermal edge flow is induced near the edges. This type of flow was first studied by Aoki *et al*.^13^ and experimentally confirmed by Sone and Yoshimoto^14^. The mechanism of thermal edge flow is essentially the same as for thermal creep flow in the simplified picture described above. On the other hand, even if the temperature of the boundary is uniform, the presence of a sharp edge can induce a large gradient of the temperature in the gas along the boundary, which causes a flow by the same mechanism as for thermal creep flow. If the temperatures of the two sides of a vane with a sharp edge are different, a force is exerted on it; the reverse force results in a gas motion around the vane from the cold side to the hot side. This phenomenon is known as radiometric flow and can be observed in the famous Crookes radiometer^15^, which has been at the focus of research during past^16^,^17^,^18^,^19^,^20^ and recent years^21^,^22^,^23^,^24^,^25^,^26^,^27^. Similarities and differences between radiometric flow and thermal edge flow have been investigated by Taguchi and Aoki^28^,^29^.

A traditional application of the microscale thermal creep phenomenon is the Knudsen compressor/pump which allows for gas flow without any moving parts under rarefied conditions. This device has attracted attention in the context of microelectromechanical systems (MEMS). Reynolds^30^ and Maxwell^31^ first studied thermal creep flow. Knudsen later built a molecular compressor based on thermal transpiration^32^,^33^, attaching his name to this type of device. Several variants of Knudsen pumps have been studied, with a typical pump consisting of curved or straight microchannels connecting two chambers at different temperatures^34^,^35^. Another Knudsen pump design comprises a channel with a temperature gradient applied to the wall surface^36^,^37^. Gupta *et al*.^38^ improved the Knudsen pump design by using microfabrication techniques for a multistage pump for vacuum pumping applications. Yet another variant termed accommodation pump has been introduced by Hobson^39^, where the design consists of two channels with dissimilar surface properties connected to form a U-shape tube. As the bottom half of the tube is held in a cold reservoir, and thermal creep pushes the rarefied gas in the section with rough walls upward more strongly than in the smooth section, resulting in a net flow through the channel. The efficiencies of various configurations of accommodation pumps were evaluated by Hudson and Bartel using DSMC^40^.

To avoid large temperature gradients in the wall material and to limit the required temperature span, a new configuration for a Knudsen pump was proposed by Donkov *et al*.^41^. A ratchet channel is used with the temperature gradient between opposing walls. Mixed diffuse and specular reflection boundary conditions are applied along the ratchet surface. Based on kinetic theory, an analytical solution has been found for the net force between the walls in the free molecular flow regime. A significant gas mass flux along the channel has been demonstrated at large but finite Knudsen numbers. In a recent study, Wang *et al*.^42^ investigated thermally and pressure driven gas flows using the unified gas-kinetic scheme^43^ in a ratchet channel with a different patterning of the specular and diffusively reflecting patches on the ratchet surface. In a variant of this arrangement, Chen *et al*.^44^ considered two facing isothermal ratchet surfaces held at different temperatures, but with diffuse reflection boundary conditions everywhere. Their simulations were performed in the slip flow regime based on the Navier-Stokes equations with velocity slip and temperature jump boundary conditions and showed that the suggested pump could work even when all surfaces are diffusive. These studies have been extended to the transition flow regime using DSMC simulations^45^. In this regime, thermal gradients at the tips of the ratchet induce an edge flow, predominantly flowing along the steeper edge of the ratchet^46^. This induces a vortical flow in the valleys between the ratchets, and by suitably adjusting the offset between the upper and lower ratchet surface a net flow along the channel is obtained. It was shown that reducing the momentum accommodation coefficient along the inclined walls of the ratchet results in a huge increase of the flow rate compared to the situation with diffuse reflection everywhere^45^.

The first motivation of the current research is to provide a detailed description of the flow induction mechanism in the ratchet pump suggested by Donkov *et al*.^41^. We will show that even though the nature of the flow in this configuration bears some similarity with thermal edge flow in the case of purely diffuse boundaries, the working principle of this pump with mixed specular and diffusive walls is more closely related to the flow around the vanes of a Crookes radiometer. Direct simulation Monte Carlo (DSMC) results for the force on the channel walls are compared with analytical results for large Knudsen numbers and with an estimate by Einstein at small Knudsen numbers. Secondly, a major motivation of this work is to study the pumping performance that can be reached by tailoring the distribution of accommodation coefficients at the walls, following up on the observation reported in ref. 45 that lowering the accommodation coefficient along the inclines tremendously increases the mass flow. For this purpose, we study the extreme case of specular reflection at the inclines. This assumption is motivated by recent results^47^,^48^,^49^,^50^, demonstrating that accommodation coefficients between 0.1 and 0.4 can be reached in practice with specific materials. The effect of applying different accommodation coefficients at the inclined wall segments on the pumping performance is also reported. The effect of geometrical parameters such as height, width and periodicity on the flow rate is investigated in detail, and optimum working conditions for the pump are recommended.

## Problem description

**Figure 1:**
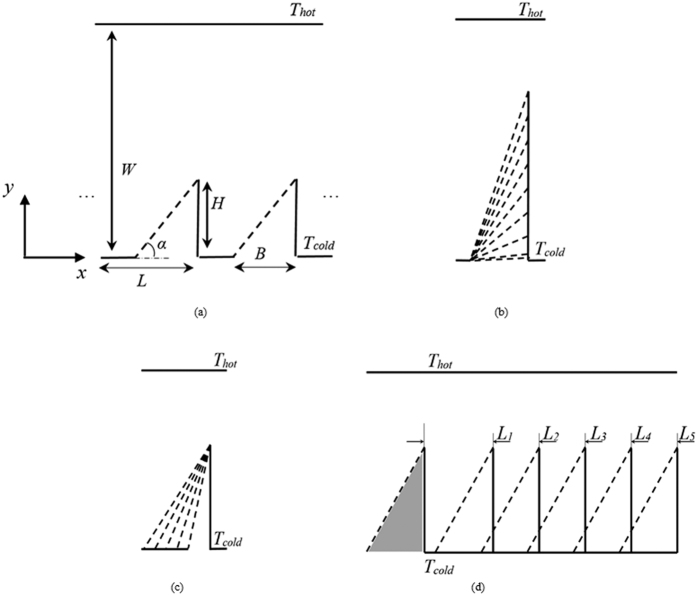
(**a**) Schematic of the channel geometry considered consisting of two surfaces with different temperatures. (**b**) Variation of the height *H* at constant *L* and *B*. (**c**) Variation of the ratchet baseline *B* at constant *H* and *L*. (**d**) Variation of the periodicity *L* at constant *H* and *B*. Tableaus (**a–c**) are drawn to scale with the variations used in this work.

Consider nitrogen gas flowing through the ratchet channel shown in Fig. 1a. A two-dimensional situation is assumed, i.e. the geometry is assumed to be infinitely extended in a direction normal to the projection plane shown in Fig. 1a. While the diffusely reflecting upper wall is unstructured, the bottom surface displays a ratchet pattern of period *L* with ratchets of height *H* and baseline width *B*. As our base geometry we consider a channel of width *W* = 400 nm and a ratchet geometry with ratios of *L*/*W* = 5/16, *B*/*W* = 1/4 and *H*/*W* = 0.43 such that α = 60° 41. If not stated otherwise, these parameters were used in the simulations. The diffuse reflection boundary condition is applied along all walls indicated by solid lines, while the specular reflection boundary condition is applied along the inclined wall segments drawn by dashed lines, if not stated otherwise. We consider the effect of changing various geometrical parameters such as the ratchet height. Starting from the base geometry, the parameters of the ratchet geometry are varied in three ways: i) the ratchet height *H* is varied at constant 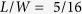 and *B*/*W* = 1/4 within the range *H*/*W* = 0 … 0.7, changing the angle *α* between 0° and 70° (Fig. 1b); ii) The width *B* of the ratchets is changed at constant 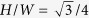 and period *L*/*W* = 5/16 within 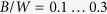, corresponding to a variation of the angle *α* between 77° and 55° (Fig. 1c); iii) The period *L* is varied at fixed 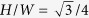 and *B*/*W* = 1/4, such that 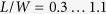 (Fig. 1d). The different versions shown in Fig. 1b to d indicate the geometries considered in the simulations. The temperature of the bottom wall is set to*T*_*cold*_ = 300*K*, and the difference between the temperatures of the top and bottom wall is chosen asΔ*T* = *T*_*hot*_ − *T*_*cold*_ = 300*K*.

## Flow Mechanism

### A Molecular Description

**Figure 2:**
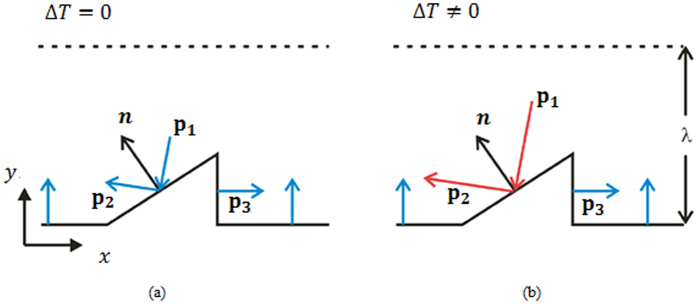
Reflection of molecules at the lower surface at (**a**) Δ*T* = 0 and (**b**) Δ*T* ≠ 0. ***p***_1_ indicates the net direction of the incoming molecules, ***p***_2_ the net direction of the reflected molecules, and ***p***_3_ the net momentum flux of the molecules reflected at the inclined wall.

To understand how the investigated thermally driven pump shown in Fig. 1a works, consider Knudsen numbers slightly smaller than 1 and ratchets of a height substantially smaller than the channel width. Also, we assume that the spacing between two neighboring ratchet fins is so large that their interaction can be neglected. Here, the Knudsen number is defined as the ratio between the molecular mean-free-path and the average distance between the upper and the lower wall. Consider a part of the lower surface, as sketched in Fig. 2. All sections of the surface exhibit a diffuse‐reflection boundary condition, apart from the inclined part of the ratchet at which specular reflection occurs, as discussed earlier.

Above the surface there is a Knudsen layer of a thickness corresponding to the mean‐free path *λ*, in which collisions between molecules can be neglected. Only once molecules reflected from the surface reach the dashed horizontal line, they can collide with other gas molecules, which is of course a simplifying assumption. Whether or not the gas volume above the dashed line can be set into motion depends on the momentum transfer between the gas and the solid surface. The gas can only be set into motion if a net momentum transfer in *x*-direction occurs upon interaction with the wall. We thus consider the bulk gas above the dashed line to be at rest and to have a phase‐space distribution not too far away from a Maxwell distribution. For this reason, it is expected that the molecules leaving the bulk of the gas in the direction of the surface do not have a preferential direction and in particular do not carry a net *x*-momentum towards the wall. The net transfer of *x*-momentum between the bulk gas and the surface is thus solely determined by the molecules reflected from the surface. Figure 2 indicates the average directions of the molecules reflected from different sections of the surface. The horizontal sections exhibit a diffuse reflection boundary condition, so on average the molecules are reflected normal to the wall segments and do not carry a net *x*-momentum. The vertical side wall of a fin also reflects diffusely, therefore on average the reflected molecules travel in the positive *x*-direction and they carry a net *x*-momentum. The inclined side wall of a fin reflects specularly, therefore the net direction of the reflected molecules depends on the net direction of the incoming molecules. Assume that the net direction of the incoming molecules is as indicated by the vector **p**_1_ in the figure above. Then the net direction of the reflected molecules is along the vector denoted as **p**_2_. On the left‐hand side of Fig. 2, the velocity vectors are drawn for a situation in which no temperature gradient is applied. The axial momentum introduced into the bulk gas per unit time is given by the sum of the *x*-components of **p**_2_, representing the net momentum flux of the molecules reflected at the inclined wall, and **p**_3_, the net momentum flux of the molecules reflected at the vertical wall. The second law of thermodynamics dictates that in the case Δ*T* = 0, this sum vanishes. Otherwise, the gas above the Knudsen layer would experience a net force due to particles reflected from the surface and it would be possible to extract work simply by cooling a single heat bath.

For comparison, on the right‐hand side of the figure above the same situation is sketched for Δ*T* ≠ 0. In that case the molecules originating from the bulk gas again have a phase‐space distribution resembling a Maxwell distribution, but this time with a higher temperature than that of the wall. Apart from the difference that –on average– the molecules originating from the gas above the dashed line have a higher velocity than in the isothermal case, the trajectories of the molecules within the Knudsen layer are identical in the isothermal and in the non‐isothermal case. Specifically, this means that the momentum flux vectors depicted in the figure above are identical in the two cases, with the only difference that the lengths of **p**_1_ and **p**_2_ increased in the non‐isothermal case. Note that the molecules reflected at the specular segment do not thermalize with the wall and maintain their incoming energy, whereas the diffusely reflected molecules thermalize with the wall. From the forgoing we conclude that while the *x*‐components of **p**_2_ and **p**_3_ balance in the isothermal case, a net momentum in negative *x*‐direction is introduced into the gas in the non‐isothermal case, owing to the fact that the length of **p**_2_ has been increased compared to the isothermal case. Therefore, the gas is set into motion, and a channel bounded by a ratchet surface with a specular‐reflection boundary condition at the inclined segments may serve as a pump.

As a third case, consider the situation where all segments of the structured wall are diffusely reflecting at uniform temperature. The fact that at the inclined wall the outward flux of momentum per unit area has a smaller *x*-component than on the vertical wall, this is compensated by the correspondingly larger area of the inclined segment such that again the *x*‐components of **p**_2_ and **p**_3_ balance. Thus, irrespective of the temperature of the bulk gas above the Knudsen-layer, there is no net momentum exchange in *x*-direction between the gas and the fully diffuse isothermal wall.

This qualitative reasoning can be made quantitative for the idealized case in which the edge of the Knudsen-layer is replaced by a diffusely reflecting wall at fixed temperature, and collisions between gas molecules are neglected. This situation was considered by Donkov *et al*. in refs 41 and 51. In these cases, the momentum transfer between the two walls can be evaluated analytically, and an explicit expression for the force on the walls was obtained. The result confirms the above reasoning, leading to a vanishing axial momentum transfer between the two walls for a diffusely reflecting isothermal lower wall and a non-vanishing transfer for a lower wall with mixed specular and diffuse reflections, as long as the lower wall has a temperature which is different from the upper wall.

### Radiometric Flow

In the argument above we have assumed that the height of the ratchet structures is of the order of the mean free path or smaller and that molecules impinging onto the lower wall from the edge of the Knudsen layer have a homogeneous temperature. A very different situation arises for much smaller mean free paths, when the temperature in particular at the tip of a fin becomes very inhomogeneous due to the mixed diffuse-specular boundary conditions on its different sides. In this case molecules impinging onto the tip from the warmer side will impart a larger momentum than on the colder side, resulting in a net force on the ratchet in a direction opposite to the temperature gradient. Due to momentum conservation an equal but opposite force acts on the reflected gas molecules, such that at finite Knudsen numbers a flow along the temperature gradient is induced at the tip of each ratchet. Note the similarity of this flow to thermal creep flow along a wall with tangential heat flux, thermal edge flow along the edge of a heated plate and in particular radiometric flow observed at the edge of a thin plate with a normal temperature gradient, respectively.

**Figure 3:**
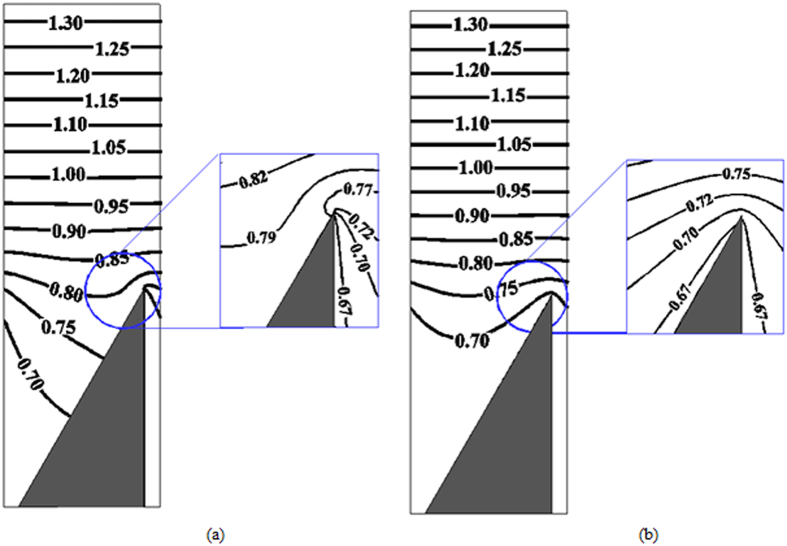
Contour lines of temperature in the continuum regime for specular, (**a**) and diffuse, (**b**) reflection on the inclined surfaces of the ratchet. The temperature field was obtained by solving the Laplace equation with the appropriate Dirichlet, Neumann and periodic boundary conditions at the edges of the domain, assuming a negligible temperature jump on walls. The zoomed frames show isolines in the near-tip regions.

To illustrate this point, we solve the heat conduction equation in the continuum regime, where the temperature is governed by the Laplace equation and temperature jumps at walls can be neglected. The corresponding isolines of temperature are shown in Fig. 3, on the left for a mixed specular diffuse lower wall and on the right for a fully diffuse lower boundary. The specular reflection renders the temperature profile strongly asymmetric around the tips of the ratchet. In this sense, the situation at the ratchet tip becomes similar to the situation encountered at the edges of the vanes of a Crookes radiometer^21^, where the light-absorbing black side heats up, while the temperature of the light-reflecting side remains closer to the temperature of the surrounding gas. On the other hand, for a fully diffusely reflecting tip the temperature profile is very symmetric, similar to the situation around a uniformly heated or cooled plate. Therefore, the main effect of using a combined specular-diffuse boundary condition is to induce wall-parallel temperature gradients in the vicinity of the tip. At finite Knudsen number, a gas flow is induced at the tip that for mixed boundary conditions resembles radiometric flow, while for a fully diffuse wall a flow similar to thermal edge flow is anticipated along the sides of a fin.

## DSMC Method and Solver

The Direct Simulation Monte-Carlo (DSMC) method is a particle-based algorithm to solve the Boltzmann equation in which every simulated particle represents a large number of real gas molecules. In DSMC, the temporal evolution of the particles within a small time interval *dt* is divided into two consecutive steps: a free molecular motion of all particles and collisions of particles localized in the neighborhoods of given points in space, i.e. in the cells of a computational grid, resulting in a change of the particle velocities through binary collisions. Here, an improved version of the original DSMC solver of the OpenFOAM software package^52^, dsmcFoamStrath, was employed^53^. The accuracy of both solvers has been suitably validated for a broad range of low-speed and high-speed rarefied flows. The collisions between particles are simulated using the Larsen-Borgnakke Variable Hard Sphere (LB-VHS) collision model. Collision pairs are selected based on the standard no time counter (NTC) collision method^54^.

As computational domain, one rectangular cell of width *L* around one fin of the ratchet is selected such that the fin lies in the middle of the domain. Periodic boundary conditions are applied in *x*-direction. The domain is divided into computational cells that serve as averaging volumes for the sampling of macroscopic flow properties. Thus, the cell size should be small enough to capture changes in the computed averages. Additionally, the cells serve as a framework to select collision pairs. In all simulations reported here, on average 50 particles per cell are employed. Transient adaptive subcells (TAS) are employed to automatically adjust the number of collision cells, i.e. cells wherein collision partners are chosen, corresponding to the local gas density gradients^55^,^56^,^57^. Subcell sizes are calculated such that on average there are two particles in each subcell. Consequently, the collision cell size becomes smaller than the mean free path, around 0.1*λ* or less for all reported cases. The time step is also set to be significantly smaller than the minimum of the mean molecular collision time and the cellular transit time^58^. As indicated above, the Knudsen number is defined as the ratio of the mean free path of the gas molecules and the characteristic length scale of the geometry, where the latter is chosen as the effective height of the channel configuration. The effective height, *W*_eff_ = *W* − (*HB*/2)/*L*, is computed as the ratio of the area of the channel and its periodicity. Using a large enough number of particles and computational cells, as well as running the simulations for a sufficiently long time on 80 CPU (Intel Xeon E5-2670 and E5-2680-v3) cores, results in reducing the statistical errors of the DSMC simulations.

## Results and Discussion

For the purpose of presenting the results, we define the average temperature as*T*_*av*_ = (*T*_*hot*_ + *T*_*cold*_)/2, the average density as *ρ*_*av*_ = *nm* where *n*is the initial number density of the gas, and *m* = 46.5 × 10^−27^ kg is the molecular mass of nitrogen. We also introduce the pressure scale *P*_*av*_ = *ρ*_*av*_*RT*_*av*_, where *R* is the gas constant, the volume flow rate 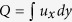, where the integral is performed on a line connecting the tip of a fin and the top wall, and the reference volume flow rate *Q*_0_ = *u*_0_*W*_*eff*_, where 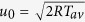 is the characteristic molecular velocity. We introduce dimensionless parameters as follows:


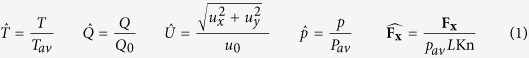


where **F**_**x**_ is the *x*-component of the force per unit length in *z*-direction on some element of the geometry. The Knudsen number is defined as


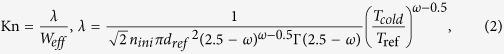


where in the variable hard sphere (VHS) collision model for nitrogen the parameters *ω* = 0.74, *d*_*ref*_ = 4.17 × 10^−10^ m, and *T*_ref_ = 273K have been used, and Γ is the gamma function.

### Temperature, pressure and velocity field

**Figure 4:**
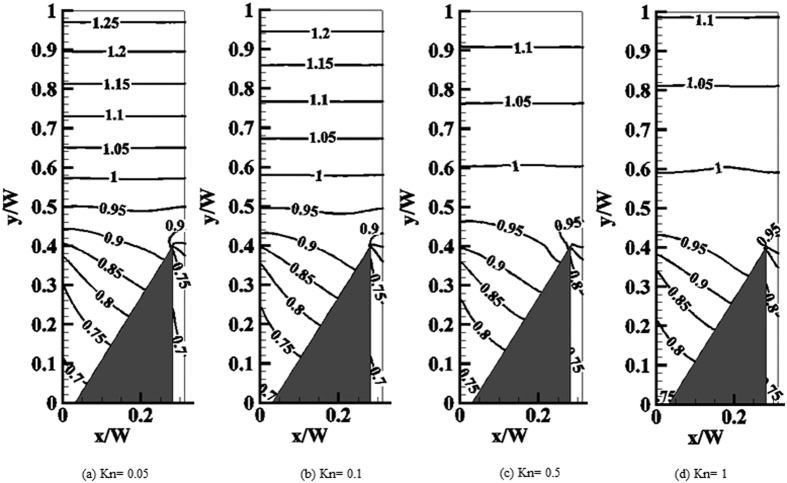
Isolines of the normalized temperature at different Knudsen numbers.

First, we focus on the base ratchet geometry (*W* = 400 nm, *L*/*W* = 5/16, *B*/*W* = 1/4, *H*/*W* = 0.4 with* α* = 60°) at different Knudsen numbers. Figure 4 shows isolines of the normalized temperature in a domain of periodicity *L* around a ratchet. For all cases considered, the normalized isoline of unit temperature is approximately parallel to the upper wall. This isoline can be found at about *y* = 0.6, above which the temperature gradient is spatially almost constant. With increasing Knudsen number the temperature jumps at the diffuse walls increase, reflected by the wider spacing between isolines. Note, however, that the temperature profile around the tip does not change drastically with Kn, apart from a slight shift towards larger values. In particular, it closely resembles the temperature profile obtained in the continuum regime, Fig. 3a.

**Figure 5:**
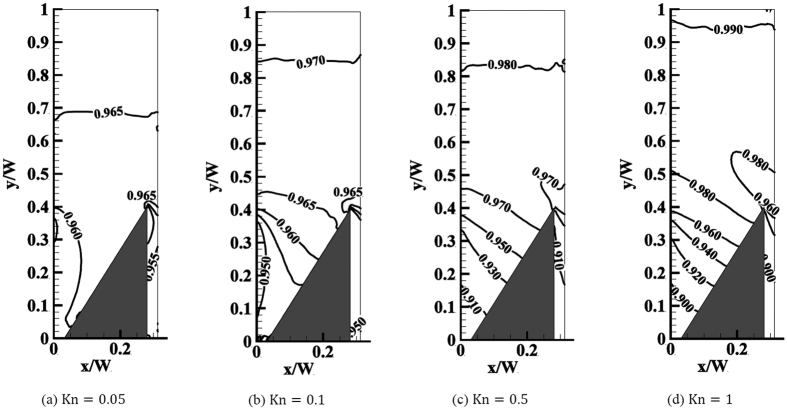
Isolines of the normalized pressure at different Knudsen numbers. For Kn ≤ 0.1 the spacing between isolines is 0.005, while for larger Kn the spacing is between 0.01 and 0.02 at the specular side of the fin.

Contrary to the temperature, the pressure distribution in the domain is altered significantly with Kn, as shown in Fig. 5. First, note that in all cases the pressure is comparatively homogeneous in the region above the ratchet. For small Kn, this homogeneous region extends far into the space between the fins, with an inhomogeneous region predominantly at the upper edge of the diffuse side. This indicates that for small Kn only the tip region of the ratchet contributes significantly to the force on the structure. With increasing Kn, this inhomogeneous region extends further into the space between fins, while simultaneously the difference in the normalized pressure on opposite sides of a ratchet fin increases. Note that in all cases, similarly to the temperature isolines in Fig. 4, the isolines of pressure tend to converge at the tip on the diffuse side of the fin, indicating strong gradients of pressure in the tip region. Judging from the pressure alone, the gas exerts a net force on the ratchet in positive *x*-direction, which by momentum conservation must be balanced by a corresponding force on the gas phase in negative *x*-direction, leading to a flow along the channel.

**Figure 6:**
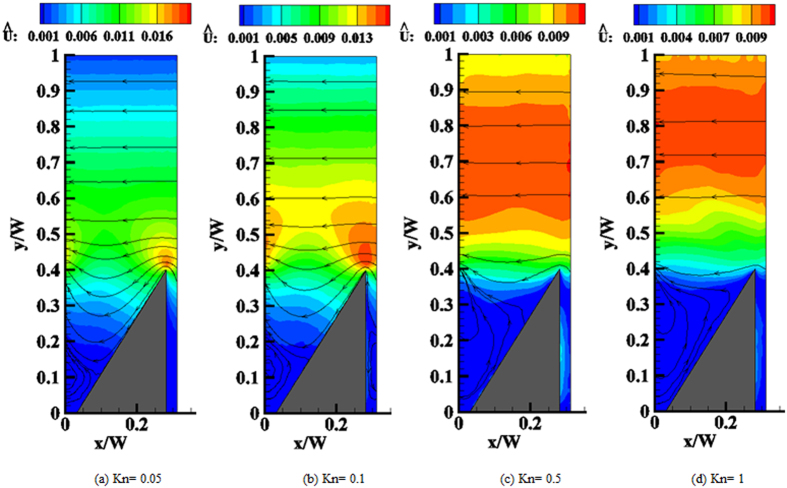
Isolines of the normalized velocity magnitude and streamlines at different Knudsen numbers.

Velocity streamlines plotted over iso-contours of flow speed are depicted in Fig. 6. The flow is induced in the negative *x*-direction, as expected from the momentum exchange with the ratchet. At low Knudsen numbers the maximum velocity occurs within a region extending roughly one mean free path directly above the tip of a fin. In this case the velocity in the open channel region above the ratchet decreases almost linearly towards the upper wall such that the velocity profile resembles a Couette flow driven by the lower surface. As Kn increases, the region of maximum velocity moves farther away from the tip until it fills almost the entire width of the open channel. In this regime the flow profile in this section nearly resembles a plug flow due to the large velocity slip at the upper wall.

For all Knudsen numbers, a vortex appears in the trough between two fins, filling it almost completely when the mean free path is of the order of the ratchet height. Note that such a vortex also appears in the continuum limit for low Reynolds number flow near a sufficiently sharp corner^59^,^60^. Although the situation is slightly different here, with a specular sidewall of the cavity and a different driving mechanism for the flow, the mechanism of vortex generation is expected to be essentially the same.

### Force on the ratchet

**Figure 7:**
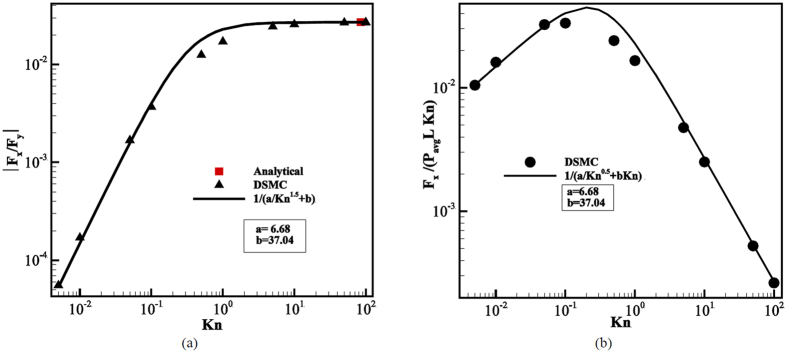
(**a**) Ratio of axial and normal force on a channel wall as a function of Kn. (**b**) Normalized axial force on a channel wall as a function of Kn.

The force on an infinitesimal wall surface element of area *dA* with outward-normal **n**_***j***_ reads d**F**_**i**_ = −*p*_*ij*_**n**_***j***_*dA*, where *p*_*ij*_ is the molecular momentum flux tensor (or pressure tensor) in the fluid. The force on any wall of the channel is thus obtained by integrating the force density over its entire surface. By Newton’s third law the net force on the opposing wall is of the same magnitude but points in the opposite direction. In Fig. 7a the ratio of forces in *x*- and *y*-direction over one entire wall boundary is shown. It appears that for small Kn this ratio scales as ~Kn^1.5^, while it saturates at a constant value for large Kn. The solid line interpolates between these two regimes using a best fit to a function *ϕ*(Kn) = [*a* + *b*/Kn^1.5^]^−1^.

Experimentally, the Knudsen number is usually changed by varying the pressure, since Kn ∼ 1/*p* at constant temperature. To directly compare the magnitude of forces on the walls measured in corresponding experiments it is thus advantageous to use the pressure scale *P*_*avg*_*L*Kn for normalization. This is done in Fig. 7b, where the normalized force in *x*-direction on the lower wall is shown for various values of Kn. Here it becomes apparent that the force maximum in experiments is expected close to Kn = 0.1. Note that since *F*_*y*_ ∼ *p* the corresponding functional dependence is 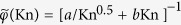, representing the solid fitting curve.

To better understand the dependency of the forces on Kn, we first turn to large mean free paths. In ref. 41, Donkov *et al*. calculated the forces on the walls in the collisionless regime and found (as above, ***F***_***i***_ is the force per unit length in *z*-direction in a periodic domain of length *L*)


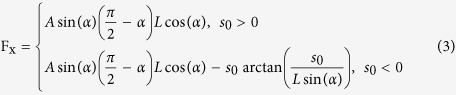



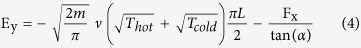


Here 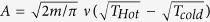, and *s*_0_ is the projection of the tip of a fin onto the incline of its neighboring fin. Note that both F_x_ and F_y_ are proportional to the particle flux density at the wall, *ν*, i.e. the number of particles colliding with the wall per unit area and time, which itself is proportional to the average particle density in the domain. Since all other factors in Eqs (3) and (4) are purely geometric or dependent on the wall temperatures, the ratio of the forces thus is independent of the particle density. The red square in Fig. 7a indicates the prediction from the analytical relations at the collisionless regime. A good agreement with the current DSMC result is observed.

For a Crookes radiometer at variable Knudsen number, Selden *et al*.^21^ were successful in qualitatively describing the force on a plate of area *A* and temperatures *T*_−_ and *T*_+_ on its left and right sides by adapting the expression,


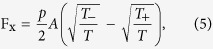


valid in the free molecular regime, by assuming that at finite mean free path in the gas phase only a region of size *λ* at the edge of the plate with perimeter 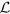 contributes to the force, replacing the full area *A* with an effective area 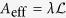. Qualitatively, a similar temperature dependence can be expected for the force on a fin of the ratchet for large opening angles *α*, although here the wall temperature is not specified as boundary condition on the specular side. However, expanding the temperature profile as *T*_±_ ≈ *T*(*x* ± *λ*) ≈ *T* ± *λ* *dT*/*dx* around the tip, Eq. (5) becomes


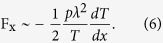


Note that this expression agrees with estimate by Einstein for the radiometric force on a plate in a temperature gradient^18^. This is no coincidence of course, as both estimates rely on the same principles, i.e. the momentum transferred to the wall by gas molecules originating from regions of different temperatures and the assumption that only the edge of the plate contributes. As we have seen in Figs 3 and 4, a strong thermal gradient also appears at the tip of a fin with mixed specular-diffuse boundary conditions. For large angles *α* we can estimate the temperature gradient at the tip along the lines of Sone^61^ and Taguchi and Aoki^28^, who estimate *dT*/*dx* ~ *λ*^−0.5^ at the edge of a plate. Combining these results, we find the dependence F_x_∼  *nλ*^1.5^ ∼ *λ*^0.5^, which is indeed the scaling observed for small Kn in Fig. 7b.

### Knudsen number effects on the volume flow rate

**Figure 8:**
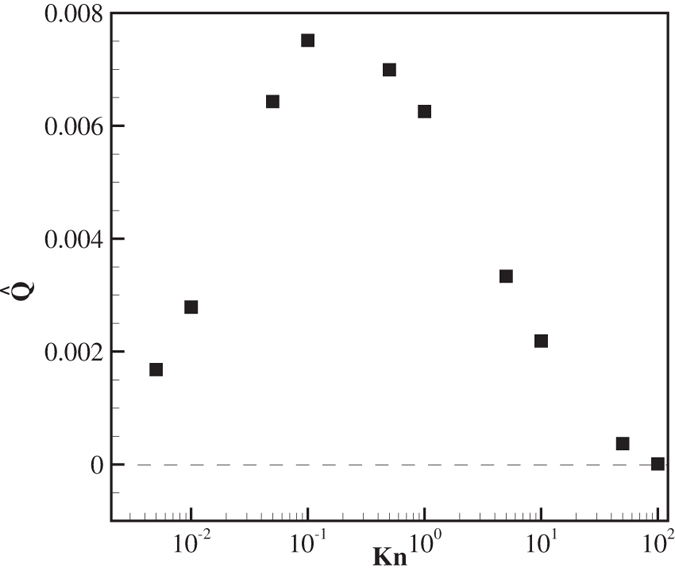
Variation of the normalized volume flow rate with Kn.

Figure 8 shows the variation of the fluid volume flow rate with the Knudsen number. The volume flow vanishes in the continuum limit Kn → 0. After an increase up to Knudsen numbers of about 10^−1^, it decreases with increasing Knudsen number in the transition regime. On dimensional grounds, for small Knudsen numbers we can assume that the velocity scales like **u** ~ **F**_**x**_*H*/*μ*, where **F**_**x**_ is the axial force per unit area, *H* is the ratchet height and *μ* is the gas viscosity. Since the gas viscosity is independent of pressure, a similar scaling for **u** and the volume flow rate as for the force can be expected at small Kn. At some intermediate point the volume flow rate reaches its maximum, which, according to the above argument for the velocity dependency on the axial force, is expected to occur where the force is at its maximum, i.e. at Kn ~ 0.2.

### Variation of the ratchet height

**Figure 9:**
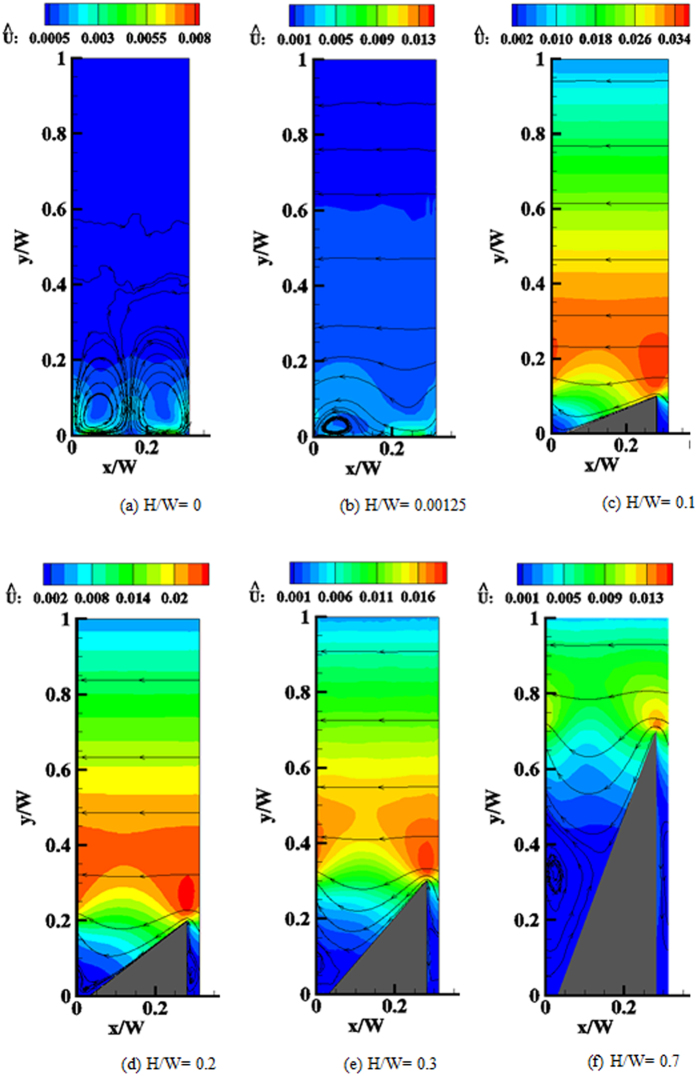
Effect of the ratchet height variation on the velocity magnitude contours and *streamlines*, Kn = 0.1.

So far we have considered a fixed geometry at variable Kn. Here, we consider the effect of changing the geometrical parameters on the pumping characteristics. First, the influence of the ratchet height variations on the flow field is investigated at Kn = 0.1 and 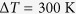. At *L*/*W* = 5/16, 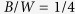, we considered *H*/*W* = 0, 0.00125, 0.0125, 0.025, and *H*/*W* = 0.1 − 0.7 with an increment of 0.1. In Fig. 9 the velocity magnitude contours and streamlines for six typical heights are shown. *H*/*W* = 0 corresponds to a lower flat wall with alternating patches of specular and diffuse reflection, the specular patch length is indicated with an arrow.

In that case two counter rotating vortices over each of the specular patches appear. The cause for the pairwise vortex formation is essentially the same as in the case of thermal edge flow. The temperature over the specular patch is higher than over the diffuse patch. Thus, molecules impart a net transverse momentum on the diffusely reflecting patch on both of its edges, pointing inward to the patch. By the same reasoning as for thermal edge flow, a gas flow is induced from low to high temperature. The symmetry of the situation dictates the two-vortex-structure shown in Fig. 9a, with no net flow along the channel. At *H*/*W* = 0.00125, Fig. 9b, this symmetry is broken and a net flow along the channel is induced by the stronger flow over the tip of the fin, which, however, is still partially counterbalanced by the thermal edge flow at the left edge of the incline. With a further increase of the fin height to *H*/*W* = 0.1, the flow over the tip of the ratchet further increases in strength, dominating the flow such that it remains attached to the inclined specular wall. However, analogous to what can be seen in Fig. 6, when further increasing the ratchet height a vortex appears in the trough between two fins similar to Moffatt vortices observed for low Reynolds number flow near sharp corners^59^,^60^. Note that the largest velocities are observed for relatively small ratchet angles. Also note that again for a wide range of angles the velocity in the channel closely resembles a Couette flow profile in the open section extending roughly from one mean free path above the ratchet towards the upper wall.

**Figure 10:**
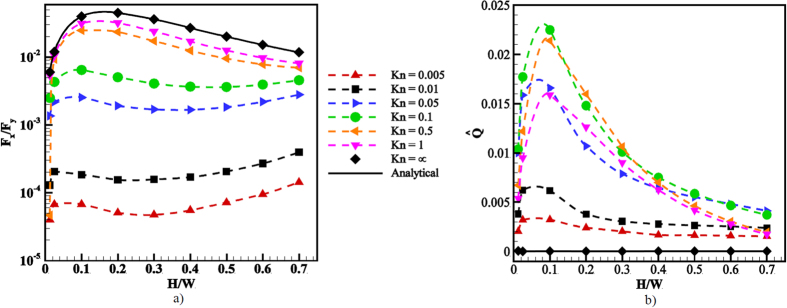
Variations of force and volume flow with the height of the ratchet for different Knudsen numbers.

Figure 10 shows the corresponding dependence of the force and volume flow on the ratchet height for various Knudsen numbers. In the left frame we again show the ratio F_x_/F_y_ of axial and normal forces on the walls. We compare the simulation results in the collisionless regime with the analytical expressions for the forces, Eqs (3) and (4), demonstrating an excellent agreement. For large values of the Knudsen number, the force ratio has a single maximum at *H*/*W* = 0.2 and drops off sharply for smaller values of the ratchet height. As Kn decreases, this maximum is shifted to smaller ratchet heights, and the curves display a positive slope for large ratchet heights. The appearance of the latter maximum can be attributed to the closer proximity to the upper wall and the corresponding increase of the thermal gradients at the ratchet tips, driving the flow. These general trends are also reflected in the volume flow rate along the channel shown in the right part of Fig. 10. Note that the maximal volume flow rate is attained in the interval 0.1 < Kn < 0.5 for *H*/*W* ≈ 0.1, corresponding to an angle *α* ≈ 20°. Also note that the volume flow for this case is by more than a factor 2 larger than for the case *α* = 60° discussed so far.

### Variation of the fin width and spatial period

**Figure 11:**
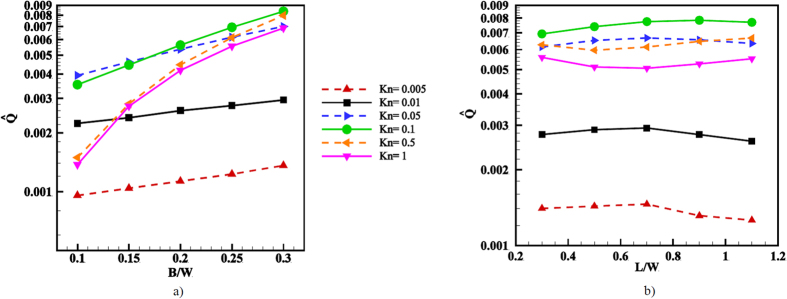
(**a**) Variation of the normalized volume flow rate with the fin width for different Knudsen numbers. (**b**) Variation of the normalized volume flow rate with the spatial period for different Knudsen numbers.

The effect of the fin width *B* on the volume flow rate, again computed on a line connecting a tip of a fin to the top wall, is shown in Fig. 11a. The width was varied as *B*/*W* = 0.1, 0.15, 0.2, 0.25, 0.3 at a constant periodicity and height of *L*/*W* = 5/16 and 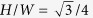 which corresponds to a variation of the ratchet angle *α* between 77° and 55°. The dependency of the volume flow rate on the angle changes with the Knudsen number. In particular, only a weak dependency is found in the slip flow regime up to Kn = 0.01, while for larger Kn the dependence becomes stronger. However, for all Kn the trend shows that a smaller ratchet angle *α* leads to a better performance and that, in particular in the transition flow regime, a large opening angle can lead to a marked decrease in the volume flow rate.

In Fig. 11b the effect of the spatial period of the ratchet pattern on the volume flow rate is considered. At 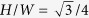 and *B*/*W* = 1/4, cases with a periodicity of *L*/*W* = 0.3, 0.5, 0.7, 0.9, 1.1 were simulated. As can be seen, for all Kn the obtained volume flow rate does not strongly depend on this parameter. However, for Kn ≤ 0.1 a maximum is reached within the parameter range considered, with a decreasing flow rate for larger fin spacing. A similar maximum is expected for larger Kn at a value of *L*/*W* slightly outside the range considered, which we were forced to limit to *L*/*W* ≤ 1.1 due to the increasing computational cost of large domain sizes.

### Dependency on the temperature difference

The energy source that drives the fluid is the heat-current, *q*_*y*_, which is directed from the upper hot wall towards the bottom cold wall. In all calculations we have assumed a large temperature difference between the walls in order to obtain large values of the induced flow and a correspondingly small noise in the DSMC simulations. However, for all quantities computed so far we expect an approximately linear scaling with the temperature difference. For small Kn the temperature profile is governed predominantly by heat conduction, such that thermal gradients are proportional to the temperature difference, as is the driving force for the flow, Eq. (6). Similarly, for large Kn heat is transferred ballistically between the two surfaces with few collisions between particles. The scale of momentum exchanged in a collision between particles originating from the upper and lower surface is 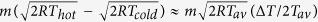, suggesting that to a good approximation the net momentum transferred to the gas phase, leading to a net flow, scales linearly with the temperature difference. To corroborate this hypothesis, we have computed the volume flow rate for values 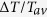 up to 0.75. Within this range, to a good approximation a linear scaling with the temperature difference is found.

### Effect of the surface accommodation coefficients

**Figure 12:**
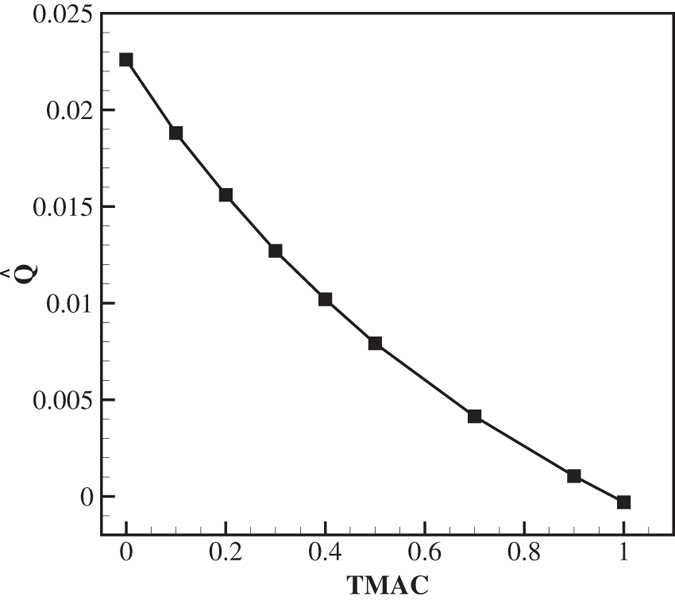
Normalized volume flow rate in the channel as a function of the accommodation coefficient at the inclines of the ratchet surface.

To estimate the effect of a departure from the idealized condition of specular reflection at the inclined wall on the pumping performance, we consider the reflection properties for this surface as specified by a Maxwell-type boundary condition with a transverse momentum accommodation coefficient (TMAC) between 0 (fully specular) and 1 (fully diffuse) at the optimum working condition and geometry, i.e. Kn = 0.1, *H*/*W* = 0.1, *L*/*W* = 0.3125, *B*/*W* = 0.25. Figure 12 shows the normalized volume flow rate as a function of the TMAC. There is almost no flow in the limit of a fully diffuse ratchet surface, and as the inclined wall becomes more specular, the volume flow increases, attributed to the increasing wall-parallel temperature gradients around the tips of the fins. This supports the key hypothesis of this work: Tailoring the momentum accommodation coefficient at the surface of a ratchet channel is of paramount importance when attempting to maximize the flow rate in thermally driven pumping.

### Concluding Remarks

While thermally-induced flow in ratchet channels has been identified as a novel and promising pumping principle for gases, an explanation of the underlying mechanism has not been given up to now. In this paper, we have shown that in the practically most relevant regime of moderate Knudsen numbers the working principle of this ratchet-type pump is similar to that of radiometric flow at the vanes of a Crookes radiometer. In particular, we emphasize that in order to induce a substantial thermal flow, large wall-parallel temperature gradients in the gas in the vicinity of the tips have to be induced. Instead of enforcing this condition by using different surface temperatures (similar to a Crookes radiometer), the pump described here benefits from different surface accommodation coefficients, which in turn induces a flow over the tip from the diffuse cold side to the hot region over the specular surface. Consequently, the heat flux between the upper and lower walls is accompanied by a mass flux from right to left in the intermediary gap. The force on the ratchet follows Einstein’s theory for radiometric flows, and bridging functions for the whole range of Knudsen number were suggested. The maximum force on the channel walls and the maximum volume flow rate occur between the slip flow and the transition flow regimes. A variation of the geometric parameters shows that the volume flow rate along the channel is strongly influenced by the height of the fins and their inclination angle with respect to the baseline. The maximum volume flow rate is attained for relatively small fins with a normalized height of about 0.1, corresponding to an inclination angle of the specular wall of approximately 20°. The volume flow rate is influenced little by the spatial period of the structured surface for the considered parameter range. Finally, it has been verified that the net volume flow rate along the channel depends approximately linearly on the temperature difference between the hot and cold walls.

For the Knudsen pump studied here, an alternating pattern of fully diffusely and ideally specularly reflecting wall segments was considered. Many surfaces have accommodation coefficients close to unity such that diffuse reflection poses a realistic approximation in most cases. On the other hand, special surfaces with accommodation coefficients between 0.1 and 0.4 have been reported, opening up the possibility that a surface with reflection properties close to the ideal situation of specular reflection considered for the inclines can indeed be fabricated.

The main benefit of using the alternating diffuse-specular wall pattern is the generation of a wall-parallel temperature gradient at the tips of the ratchet. Note, however, that since molecules do not thermalize on the specular parts of the surface, this also leads to a reduced heat transfer compared to a ratchet pump with two opposing diffusively reflective walls, with a positive effect for the overall efficiency. Also, since no tangential momentum is transferred to specular walls, this configuration results in reduced viscous dissipation. Finally, in view of the Couette-like velocity profile with a maximum at the ratchet surface visible in Fig. 9, it is conceivable that the use of two opposing ratchet surfaces could result in a significant increase of the pumping velocity compared to the configuration with only one structured wall studied here^45^.

## Additional Information

**How to cite this article:** Shahabi, V. *et al*. Thermally induced gas flows in ratchet channels with diffuse and specular boundaries. *Sci. Rep.* **7**, 41412; doi: 10.1038/srep41412 (2017).

**Publisher's note:** Springer Nature remains neutral with regard to jurisdictional claims in published maps and institutional affiliations.
